# Novel QTL for chilling tolerance at germination and early seedling stages in sorghum

**DOI:** 10.3389/fgene.2023.1129460

**Published:** 2023-03-15

**Authors:** Niegel La Borde, John Rajewski, Ismail Dweikat

**Affiliations:** Department of Agronomy and Horticulture, University of Nebraska, Lincoln, NE, United States

**Keywords:** SNP’s, sorghum breeding, QTL, GBS, chilling tolerance

## Abstract

Sorghum (Sorghum bicolor L.) a drought tolerant staple crop for half a billion people in Africa and Asia, an important source of animal feed throughout the world and a biofuel feedstock of growing importanceorghum’s originated from tropical regions rendering the crop to be cold sensitive. Low temperature stresses such as chilling and frost greatly affect the agronomic performance of sorghum and limit its geographical distribution, posing a major problem in temperate environments when sorghum is planted early. Understanding the genetic basis of wide adaptability and of sorghum would facilitate molecular breeding programs and studies of other C4 crops. The objective of this study is to conduct quantitative trait loci analysis using genotying by sequencing for early seed germination and seedling cold tolerance in two sorghum recombinant inbred lines populations. To accomplish that, we used two populations of recombinant inbred lines (RIL) developed from crosses between cold-tolerant (CT19, ICSV700) and cold-sensitive (TX430, M81E) parents. The derived RIL populations were evaluated for single nucleotide polymorphism (SNP) using genotype-by-sequencing (GBS) in the field and under controlled environments for their response to chilling stress. Linkage maps were constructed with 464 and 875 SNPs for the CT19 X TX430 (C_1_) and ICSV700 X M81 E (C_2_) populations respectively. Using quantitative trait loci (QTL) mapping, we identified QTL conferring tolerance to chilling tolerance at the seedling stage. A total of 16 and 39 total QTL were identified in the C_1_ and C_2_ populations, respectively. Two major QTL were identified in the C_1_ population, and three major QTL were mapped in the C_2_ population. Comparisons between the two populations and with previously identified QTL show a high degree of similarity in QTL locations. Given the amount of co-localization of QTL across traits and the direction of allelic effect supports that these regions have a pleiotropic effect. These QTL regions were also identified to be highly enriched for genes encoding chilling stress and hormonal response genes. This identified QTL can be useful in developing tools for molecular breeding of sorghums with improved low-temperature germinability.

## 1 Introduction

Sorghum (Sorghum bicolor L.) is the fifth most important cereal crop in the world and shows high production performance in a wide range of adverse conditions (Espitia-Hernández et al., 2020; Rashwan et al., 2021). A multi-purpose crop, sorghum has been traditionally used as grain and straw, and is also a promising crop for bioenergy production from starch, sugar, or cellulose on marginal lands with limited water and other (Teferra et al., 2019; Espitia-Hernández et al., 2020; Kimani et al., 2020; Palacios et al., 2021). It has been used as a staple for millions of people in several countries, mainly in Africa and Asia. Itis a tropical C4 grass possessing many advantageous qualities to be a designated energy crop. Sorghum has a high biomass yield, high drought tolerance, high sugar content in the stalk, a short life cycle, and high adaptability to various soils and climates. Unfortunately, to be optimally utilized in the temperate US Great Plains, sorghum must develop the ability to withstand early-season chilling temperatures. The Great Plains generally have a frost-free growing season from May through September. Sorghum must reach maturity during this brief period and be agronomically competitive with other biofuel crops.

Sorghum is susceptible to chilling injury as a tropical plant, and its development is negatively affected by temperatures of 20°C and below (Peacock, 1982; Anda and Pinter, 1994). Chilling temperature stress is typical when periods of wet and cool weather overlap with planting and is exacerbated on poorly draining heavier soils. Poor seed establishment, decreased emergence rate, decreased growth after emergence, and increased susceptibility to seedling pathogens (especially Pythium spp.) have been observed in low temperatures (Soujeole Miller 1984). Poor tolerance to cold in the early season reduces yields of both biomass and grain (Saballos, 2008). The photosynthetic machinery is susceptible to chilling under field conditions in the spring, where chilling temperatures occur along with high light intensities (Jompuk et al., 2005). Root development and architecture are crucial for chilling tolerance in sorghum (Bekele et al., 2014). Although it originates in the tropics of Africa, the remarkable scope of genetic diversity among the different subspecies has conferred an extraordinarily broad adaptability and a highly versatile range of end uses (Boyles et al., 2019). Genetic variation has been observed in early and late plantings in both fields and controlled environments (McBee and Miller, 1982; Brar and Stewart, 1994). Identifying and characterizing genetics responsible for this observed variation is critical to improving sorghum performance in temperate climates. Sowing sorghum earlier in the spring would allow for better utilization of winter moisture (Patane et al., 2006). Genetic markers associated with early season seedling emergence and germination under cold stress were studied under uncontrolled field and controlled indoor conditions (Cisse and Gebisa, 2003; Yu et al., 2004; Ejeta and Knoll, 2007; Burow et al., 2010; Bekele et al., 2014; Fielder et al., 2016; Marla et al., 2019; Schaffasz et al., 2019). These studies provided valuable insights into the chromosomal regions (QTL) underlying sorghum adaption/vigor in chilling conditions, yet some of the interpretations may be clouded by QTL-by-environment interactions (Burow et al., 2010), lack correlation to field-based screenings (Yu et al., 2004; Franks et al., 2006), and limited validation in multiple genetic materials. Therefore, the present study aimed to conduct a QTL analysis of physiological traits and identify potential candidate genes underlying chilling tolerance in sorghum seedlings under field and controlled environments. The identification of significant QTL-marker associations can be used to facilitate indirect selection for cold tolerance in sorghum breeding. To accomplish this, two segregating (for chilling tolerance) recombinant inbred (RIL) populations were evaluated for pre- and post-seedling development using genotyping by sequencing method.

Genotyping-by-sequencing (GBS) provides a low-cost high-density genotyping approach that avoids ascertainment bias (Elshire et al., 2011). Genotyping-by-sequencing has been used to increase genomic resources rapidly in many crops (Poland et al., 2012; Hu et al., 2015; Lasky et al., 2015; Felderhoff et al., 2016; Wallace and Mitchell, 2017). In sorghum, large-scale GBS data were generated to characterize genomic diversity patterns and map genomic loci underlying complex trait variation (Lasky et al., 2015; Yu et al., 2016; Bouchet et al., 2017; Hu et al., 2019).

The present study aimed to: 1) investigate the physiological responses of sorghum seedlings responses to chilling stress using two strongly segregating (for chilling tolerance) recombinant inbred (RIL) populations. 2) The identification of significant QTL-marker associations of physiological traits and identify potential candidate genes underlying chilling tolerance in sorghum seedlings under field and controlled environments.

## 2 Materials and methods

### 2.1 Environmental conditions

Typical sorghum planting dates in Nebraska are mid-to-late May when soil temperatures average about 20°C. Early season sorghum planting was during mid-April at Lincoln and Havelock. The cumulative precipitation in each environment in 2014 and 2012 was higher than in the environments in 2013 (Supplementary Table S1). The environment in 2013 also experienced snowfall twice during the evaluation period. In 2013, the experimental locations had fewer days with maximum temperatures above 15°C (29 days in 2012 and 23 days in 2014, while there were 18 days in 2013).

### 2.2 Germination tests

For this study, two populations were used, CT19 X TX430 (C_1_) and ICSV700 X M81 E (C_2_) populations, respectively The variation in cold and optimal temperature seed germination of the parents and RILs, were examined according to procedures set forth by the Association of Official Seed Analysts (AOAC, 2019). Three replications of a completely randomized design of 50 seeds were imbibed on polystyrene Petri dishes (10 cm) with filter paper (Whatman No. 1) moistened with distilled water (4 mL). The seed was incubated in the dark for 7 days at optimal temperature (28°C) and 30 days at chilling temperature (10°C). Total germination was scored daily visually by the protrusion of radicle (1 mm) from the seed at 7 days for optimal conditions and 7, 15, 22, and 30 days after sowing for cold treatment. The cumulative germination data was used as input into a curve fitting module (GERMINATOR) developed by Joosen et al. (2010) to extract germinability parameters. The module determined the maximum percentage of germination (Gmax), the onset of germination, based on 4% germination (T_4_) germination speed measured as time to 50% germination (T_50_) uniformity of germination, based on the time interval between 14% and 86% seed germination (U_8416_), and the area under the curve after 336 and 720 h of imbibition under optimal and chilling respectively (AUC).

### 2.3 Field experiment

Typical sorghum planting dates in Nebraska are mid-to-late May when soil temperatures average about 20°C. Early season sorghum planting was during mid-April at Lincoln and Havelock. The cumulative precipitation in each environment in 2014 and 2012 was higher than in the environments in 2013 (Supplementary Table S1). The environment in 2013 also experienced snowfall twice during the evaluation period. In 2013, the experimental locations had fewer days with maximum temperatures above 15°C (29 days in 2012 and 23 days in 2014, while there were 18 days in 2013.

To explore the variation in chilling/optimal field emergence, 50 seeds from the parents and RILs were sown in five × .75 m plots under rain-fed conditions at Havelock and Lincoln, Nebraska, from 2012 to 2013 and 2014. Both locations were planted in mid-April, about a month earlier than the average sorghum planting time in the Great Plains. The C_2_ population was sown only in the Lincoln, NE, location in 2013 and at both locations in 2014. All but the C_2_ in the 2013 experiments were laid in an alpha lattice incomplete block design with 16 unfinished blocks of fifteen plots (16 × 15αlattice) per replication with two replications per environment. Due to limited seed, the C_2_ population was sown in an augmented incomplete block design with five blocks of 35 plots, with the parents of both people serving as controls in each block. Emergence was assessed by counting, on alternate days, the total number of plants having emerged 30 days after sowing. Emergence data was also evaluated by GERMINATOR software (Joosen et al., 2010). Maximum emergence was coded Emax. All other parameter labels remained the same.

### 2.4 Soil-based assay

Seed emergence and seedling development in soil were observed in an alpha lattice incomplete block design with two replications. Each block was planted in 4 × 200-cell cone-trainer flats (Stewart and Sons, Inc. Corvallis, OR) with one seed per cell. Each flat was divided into quarters with 50 cells per RIL. Plants were grown under 16-h days at optimum and chilling temperatures. The plants were watered every other day, and humidity was set at 70%.

The seedlings sown at optimum condition were harvested 14 days after sowing. The seedlings grown in chilling conditions were harvested 30 days post-sowing. Total emergence was recorded every other day until harvest for optimal and cold experiments. The cumulative emergence data were input into the curve fitting module developed by Joosen et al. (2010). Seedling biomass development was determined by recording stem length (STML), root length (RTL), stem fresh weight (STMWW), stem dry weight (STMDW), root fresh weight (ROOTWW), and root dry weight (ROOTDW). At 14 days after planting, ten seedlings per RIL were taken out of the soil and rinsed with water to remove soil from the root. Shoot and root were separated from one another, and roots were wrapped in a paper towel for 2 hours to absorb excess moisture before determining lengths and fresh root weight. Roots and shoots were then dried for 7 days at 85°C, after which dry weights were determined. Separated root and shoot measurements were pooled over the ten seedlings to prevent measurement errors on individual sample weights. The greenness of the third leaf was recorded as the mean of three measuring points on ten random seedlings using a SPAD-502 plus chlorophyll meter (Konica Minolta Sensing Inc., Osaka, Japan).

### 2.5 DNA extraction

Extraction of high-quality DNA from leaf tissue was carried out utilizing a modified protocol proposed by (Xin and Chen, 2012; Jiao et al., 2018). Leaf tissue was harvested from 14- day-old seedlings in the greenhouse. Approximately 1 g of fresh tissue was harvested and placed on ice in a 2 mL microcentrifuge tube. The harvested samples were transferred to a −80°C freezer overnight. The next day, the samples were transferred to a freeze dryer and allowed to lyophilize for 2 days. A 4.5 mm steel zinc-plated pellet (Daisy Outdoor Products, Rogers, Arkansas) was added to each microcentrifuge tube containing the dried leaf samples. The tubes were sealed and transferred into Tissuelyser II grinding racks. Forty-eight tubes were ground, by TissueLyser II, at 28 strokes per minute for 1 minute. Tubes were then rotated and ground for an additional minute.

To each tube, 750 µL of extraction buffer (100 mM Tris (pH8), 20 mM EDTA, 2%CTAB, 1.2M NaCl, 0.1% B-mercapoethanol) is added. Tubes were then sealed and vortexed for 20 s and then incubated at 60°C, on a dry block, for an hour. Tubes were allowed to cool on benchtop for 5 minutes then 750 µL of chloroform:isoamylalchohol (24:1 v/v) was added to each tube. Tubes were vortexed for 20 s and then centrifuged at 3000 *g* for 15 min. A new set of 2 mL microcentrifuge tubes were labeled and filled with 500 µL of the aqueous layer from centrifuged tubes. To these tubes, 1 mL of dilution buffer (100 mM Tris (pH8), 20 mM EDTA 0,2%CTAB) was added. Tubes were vortexed for 20 s and incubated at 60°C for 30 min. Tubes were then centrifuged at 3000 *g* for 13 min at 4°C. Each tube’s supernatant was carefully poured off, and 1 mL of washing buffer (30% ethanol 70% TE (10 mM Tris, 1 mM EDTA)) was mixed gently by inversion and allowed to rest for 30 min. Tubes were then centrifuged at 3000 *g* for 15 min at 4°C. The supernatant of each tube was gently poured off, and 100 µL of high salt TE (10 mM Tris (pH 8), 2 mM EDTA, 1M NaCl) and 5 µL of RNAse was added to each tube. Tubes were gently mixed by inversion and incubated for 30 min at 60°C. The content of each microcentrifuge tube was transferred to 96-well PCR plates. To each well of the PCR plate, 5 µL of MagAttract suspension G solution (Qiagen, Venlo, Netherlands) was added along with 120 µL of absolute ethanol. A silicone mat was used to seal the PCR plate. The plates were mixed by inversion and allowed to rest on the bench for 5 minutes. The plates were then placed on 96-well magnetic plate, and ethanol was removed by inverting the plate. The magnetic beads in each well were washed three times by alternatively adding 200 µL of washing buffer to each well, mixing the plate by inversion, placing a plate on a magnetic plate, and discarding the washing buffer. After the third wash, plates were allowed to air dry on the lab bench for 10 min. To each well, 100 µL of TE buffer has been added. The plates were sealed, placed in an incubator, and incubated for 5 minutes at 60°C. Plates were gently mixed by inversion and then placed on a magnetic plate, and the TE solution was then transferred to a new 96-well plate.

### 2.6 DNA quantification and quality

Genomic DNA (2 µL/sample) was quantified using Thermo Scientific Nanodrop 8,000 spectrophotometer instrument (Fisher Scientific, Waltham, MA). The quality of DNA was then examined by digesting genomic DNA (2 µL per sample) with *Hind* III restriction endonuclease. DNA pre- and post-digestion were visualized on 1% agarose gel in 1x TBE stained with ethidium bromide, utilizing *λ* standard DNA dilution series as a control. Samples were stored at −20°C until shipped to Institute for Genomic Diversity at Cornell University (Ithaca, NY).

### 2.7 Statistical analysis

Germination and emergence performance can be interpreted by extracting relative parameters from a time curve as described by El-Kassaby et al. (2008). Where the four-parameter Hill function is utilized to fit the germination/emergence data:

y=y_o+〖ax〗^b/(c^b+x^b )

Where y is the cumulative germination/emergence at x (time in hours), y0 is the *y*-intercept, greater than or equal to zero, a is the maximum cumulative germination/emergence percentage, b controls the shape and steepness of the curve and c is the time for 50% germination/emergence (T50). The initial a and c values are extracted from the cumulative germination/emergence count, and b is set to 20. Utilizing the Germinator curve fitting module (Joosen et al., 2010), five emergence/germination parameters were estimated: time to onset of germination/emergence [time to 4% germination, in hrs. (T4)], rate of germination/emergence (time to 50% germination/emergence, in hours), uniformity of germination/emergence (time between 16% and 84% germination/emergence, (U8416) maximum germination/emergence (Gmax,%) and area under the curve between time zero and time until the last sampling. Greater AUC values are indicative of an earlier and greater germination/emergence.

Descriptive statistics collected for traits from both the indoor and outdoor screenings were subjected to an analysis of variance (ANOVA) statistical analysis, to determine the significance of genotypic effect to cold tolerance. The means and significant differences of means, for each phenotype, were determined, for each individual environment, using the Agricolae package (Mendiburu and Simon. 2015), in the R statistical sotware (R Core Team, 2012). The alpha lattice incomplete block design model for each screening was:

Yijk=μ+gi+rj+〖bk〗j+ϵij

Where *µ* is the overall population mean, gi is the genotypic effect, rj is the replication effect, bkj is the random block effect nested within the replication effect, and εij is the residual effect with a ∼ N (0, σ2b). Where the genotypes were treated as fixed effects and replication and blocks were considered random effects. The augmented partially balanced incomplete block design model for the 2013 C2 screening was:

$$Yij=\left(\mu +\rho i+\tau j+\epsilon ij\;\right)\;nij$$

Where *µ* is the overall population mean, ρij is the block effect, τj is the genotypic effect, and εij is the residual effect with a ∼ N (0,σ2b). The ANOVA results were used to estimate the heritability: h^_2_ = V_l/(V_l + V_le + V_e), respectively, the estimates of the variance components of lines (genotype), line × environment interaction, and environments (Basford et al., 2004). Pearson’s correlation coefficients among the traits were calculated on a least square mean basis.

### 2.8 Genotyping-by-sequencing (GBS)

Genotyping of the C_1_ and C_2_populations, along with their progenitors, was performed by the Institute for Genomic Diversity according to the genotyping-by-sequencing (GBS) protocol described by Elshire et al. (2011). Four 96-well microtiter plates (two per population, containing 35 µL of the previously extracted DNA were sent to the Institute for Genomic Diversity at Cornell University. Where briefly the DNA samples were digested using the APeKI restriction enzyme, 96x multiplexed libraries were assembled and sequenced *via* Illumina Genome Analyzer IIx (Illumina, San Diego, CA). To extract the single nucleotide polymorphisms (SNP) genotypes, the raw reads provided by the Institute were analyzed in the TASSEL software GBS pipeline (Lu et al., 2012), 86 bp raw reads were aligned to the BTX623 sorghum reference genome (Sorghum bicolor v1.4). Loci polymorphisms were detected by comparison of consensus sequences from all samples. As RILs were utilized in the construction of the libraries, loci with heterozygotes >10% of total RIL were discarded to reduce false positive results. Only loci with less than 20% missing data were used in the mapping.

### 2.9 Linkage map construction

Linkage maps for the two populations were created using the SNP data from GBS using R/ASMap (Taylor and Rowley, 1971). The Kosambi function (Kosambi, 1944) was utilized to convert recombination fractions into centiMorgans (cM). To detect segregation distortion chi-square (χ^2^) was calculated using R/ASMap. Unlinked and highly distorted markers were excluded from the analysis. The family-wise error rate, arising from multiple *χ* two tests, was controlled with the Bonferroni correction. Linkage maps for both C_1_ and C_2_ populations were visualized graphically with Mapchart 2.3 (Voorrips, 2002).

### 2.10 QTL analysis

QTL mapping was performed on all the germination/emergence parameters, along with SPAD, root and shoot length, and fresh and dry weight of both roots and shoots. QTL mapping was based on the average phenotypic values recorded in each environment and data averaged across all environments. QTL and their positions were determined using the inclusive composite interval mapping (ICIM) function of the IciMapping 4.0 program (Li et al., 2007). A minimum logarithm of odds (LOD) of 3.0 was set to declare the significance of detected QTL. QTL with a LOD score between two and three were considered putative QTL. The “walking speed” of 1 cM and a “window size” of 10 cM were set for the ICIM. A permutation threshold of 0.01 for each trait using 1,000 permutations (Doerge and Churchill, 1996) was set. 1-LOD support intervals were determined as described by Wang et al. (2011). The percentage of variation (*R* ^2^) and the additive effects are determined at their peak LOD value for each trait. The QTL maps were graphically visualized using MapChart 2.3 software (Voorrips, 2002).

## 3 Results

### 3.1 Germination assay

The germination test confirmed that the viability of both RIL populations was relatively high; the C_1_ population had a lower germination rate (75%) than the C_2_ population (88%). Seed germination of sorghum was significantly affected by temperature; the germination decreased to 31% (C_1_) and 45% (C_2_) at 10°C (Tables 1, 2). As expected, the cold-tolerant genotypes were observed to germinate earlier (T_4_), at a faster rate, more uniform, and with a more significant percentage than the cold-susceptible genotypes. In the RIL population, observed germination (Gmax) was more critical, earlier (lower T_4_), at an increased rate (lower T_50_), and more uniform (lower U_8416_) under greenhouse conditions than in the cold room for both the C_1_ and the C_2_ populations (Table 3). The variance analysis indicated that RIL’s genotypic effect was significant in both population**.**

**TABLE 1:** Early field emergence data for C_1_ Population at Lincoln, NE and Havelock, NE during springs 2012–2014.

		2012				2013					2014					
Traits	Location	CT19	TX430	Mean ±SD	Range	h^2^ (%)	CT19	TX430	Mean ±SD	Range	h^2^ (%)	CT19	TX430	Mean ±SD	Range	h^2^ (%)
E_max_ (%)	Lincoln, NE	84.1	30.4	46.25 ± .164	0–100	79.28	20.5	11.9	18.8 ± .132	1.17–55.6	69.82	74.1	22.1	48.7 ± .244	20.1–86.1	74.90
	Havelock, NE	46.2	21.8	27.25 ± .147	0–59	66.69	32	8	18.8 ± .1320	0.0–31	74.25	19.2	10.1	15.9 ± .125	4.08–41.2	82.99
U_8416_(h)	Lincoln, NE	102.2	137.1	132.5 ± 40.57	40.2–294.8	73.15	87.23	141.9	84.9 ± 39.12	11.1–194.7	66.57	98.46	66.15	79.9 ± 57.4	0.618–255.9	75.94
	Havelock, NE	141.3	108.2	115.9 ± 48.5	56–217.5	68.54	69.5	46.6	71.2 ± 45.4	11.2–451.7	71.18	20.51	162.1	83.5 ± 106.6	11–334	79.24
T_50_ (hr)	Lincoln, NE	408.2	463.2	430.9 ± 28.87	352.6–521.5	72.19	420.7	397	414.1 ± 40.54	326–527.8	65.47	501.02	495.3	509.9 ± 157	228.3–531.4	79.24
	Havelock, NE	351.6	399.6	375.9 ± 38.2	330.9–484.4	69.77	411.6	473.3	433.2 ± 31.9	196.6–428.4	70.95	394.8	326.2	335.04 ± 157.46	8.56–538.46	76.38
T_4_ (hr)	Lincoln, NE	321.4	349.4	321.9 ± 22.73	261.1–390.2	95.30	314.16	366.88	360.2 ± 45.1	282.5–482	70.83	295.7	336.4	268.5 ± 130.4	7.04–469.19	77.13
	Havelock, NE	422.1	445.8	307.6 ± 30.56	246.8–434.6	67.03	352.4	412.9	369.1 ± 50.9	215.9–420.6	70.82	440.6	474.6	391.4 ± 53.8	227.9–484	73.36
AUC(hr)	Lincoln, NE	254.8	176.8	167.7 ± 74.3	45.3–307.9	67.19	127.1	129.3	80.5 ± 48.2	13.3–189.4	74.35	246.8	71.2	152.8 ± 116.9	3.09–427.23	81.48
	Havelock, NE	290.2	249.5	282.2 ± 44.6	167.7–423.6	66.69	61.1	51	57 ± 15.0	11.1–187.4	74.79	62.14	44.4	50.4 ± 44.04	10.5–143.4	84.95

E_max_ = maximum emergence percentage; U_8416_ = uniformity of germination, based on the time interval between 14% and 86% emergence; T_50_ = time to 50% emergence; T_4_ = time to 4% emergence; AUC, the area under curve after 720 h.

**TABLE 2:** Early field emergence data for C_2_ Population at Lincoln, NE and Havelock, NE during springs 2012–2014.

		2013					2014					2014				
Traits	Location	M81E	ISCV700	Mean ± SD	Range	h^2^	M81E	ISCV700	Mean ± SD	Range	h^2^	CT19	TX430	Mean ± SD	Range	h^2^
Emax (%)	LincolnNE.	70.8	59.7	60.9 ± 26.9	14.6–89.2	61.69%	64	40.8	57.5 ± 18.3	11.3–88.2	74.29%	74.1	22.1	48.7 ± .244	20.1–86.1	74.90%
	Havelock, NE	-	-	-	-	-	34.8	32.9	30.6 ± 14.9	1.0–54.5	66.34%	19.2	10.1	15.9 ± .125	4.08–41.2	82.99%
U8416 (hr)	Lincoln, NE	52.6	70.2	67.71 ± 42.8	46.8–111.7	59.07%	83.2	90.3	90.54 ± 37.8	8.35–170.9	66.93%	98.46	66.15	79.9 ± 57.4	0.618–255.9	75.94%
	Havelock, NE	-	-	-	-	-	128.9	183.1	174.7 ± 232.2	8.5–680.0	65.72%	20.51	162.1	83.5 ± 106.6	11–334	79.24%
T50 (hr)	Lincoln NE.	418	400.9	410.2 ± 21.9	378.8–428.7	54.31%	443.1	432.4	440.5 ± 20.8	350.8–532.9	67.76%	501.02	495.3	509.9 ± 157	228.3–531.4	79.24%
	Havelock NE.	-	-	-	-	-	340.1	401.7	362.6 ± 61.1	231.1–459.6	66.34%	394.8	326.2	335.04 ± 157.46	8.56–538.46	76.38%
T4 (hr)	Lincoln NE.	372.6	368.6	351.9 ± 38.5	348.9–375.5	57.60%	361.9	355.3	363 ± 26.7	309.6–401.7	69.76%	295.7	336.4	268.5 ± 130.4	7.04–469.19	77.13%
	Havelock, NE	-	-	-	-	-	289.7	296.5	291.3 ± 78.2	183.5–432.2	67.40%	440.6	474.6	391.4 ± 53.8	227.9–484	73.36%
AUC	Lincoln NE.	257	209.9	222.8 ± 72.3	68.0–306.7	73.66%	224.66	185.5	204.9 ± 67.2	21.7–447.2	71.96%	246.8	71.2	152.8 ± 116.9	3.09–427.23	81.48%
	Havelock, NE	-	-	-	-	-	142.4	125.7	125.1 ± 61.8	18.5–228.5	69.52%	62.14	44.4	50.4 ± 44.04	10.5–143.4	84.95%

Emax = maximum emergence percentage; U8416 = uniformity of germination, based on the time interval between 14% and 86% emergence; T50 = time to 50% emergence; T4 = time to 4% emergence; AUC, the area under curve after 720 h.

**TABLE 3:** Indoor germination data for C_1_ and C_2_ population.

		Population C1				Population C2			
		Parents		RILs		Parents		RILs	
Traits	Location	CT19	TX430	Mean ± SD	Range	M81 E	ICSV700	Mean ± SD	Range
Gmax (%)	Greenhouse	83.0	79.0	75.0 ± 11.0	10.0–100.0	86.5	90.5	60.0 ± 38.0	0.0–100.0
	Cold Chamber	44.0	20.0	31.0 ± 17.0	0.00–82.0	25.0	35.0	45.0 ± 24.0	0.0–100.0
U8416 (hr)	Greenhouse	66.5	68.8	73.9 ± 20.6	17.5–131.6	30.4	23.3	28.0 ± 16.3	2.0–88.4
	Cold Chamber	37.2	200.9	166.8 ± 67.1	16.5–544.5	199.9	112.6	134.7 ± 75.2	13.4–322.9
T50 (hr)	Greenhouse	126.4	140.0	127.2 ± 19.8	18.6–195.4	145.7	150.0	117.8 ± 53.3	0.0–184.2
	Cold Chamber	300.7	412.9	392.3 ± 56.9	52.6–622.2	549.6	450.5	455.8 ± 69.9	55.4–570.5
T4 (hr)	Greenhouse	76.9	88.5	75.5 ± 18.5	18.2–161.9	125.4	125.4	100.2 ± 36.4	13.0–147.8
	Cold Chamber	267.2	260.2	297.2 ± 57.5	55.3–602.7	530.8	354.8	350.8 ± 80.7	75.9–530.8
AUC	Greenhouse	167.5	55.1	93.1 ± 57.5	0.00–277.2	132.4	133.3	116.0 ± 46.7	0.0–170.9
	Cold Chamber	554.7	517.4	493.7 ± 75.4	70.5–673.2	62.5	112.4	158.9 ± 81.6	2.3–379.6

Gmax = maximum germination percentage; U8416 = uniformity of germination, based on the time interval between 14% and 86% emergence; T50 = time to 50% emergence; T4 = time to 4% emergence; AUC, the area under curve after 360 (greenhouse) and 720 h (Cold Chamber).

### 3.2 Field screening of emergence

The mean performance of field emergence rates for both the C1 and C2 populations under chilling and optimal conditions are presented in Tables 1, 2. In both population, the cold-tolerant parent (CT19 and ISCV700) emerged earlier (lower T4) and had a faster rate of germination (lower T50) across all locations and years (Table 1). In the C1 population, seedling emergence was greater (↑ Emax), faster (↓ T50), and earlier (↓ T4) at the Lincoln location than at the Havelock location. Generally, seedling emergence was most significant at the Lincoln location due to warmer temperatures. Still, in 2014 there was an herbicidal carryover from a previous season that reduced germination rates in the Havelock, NE, area. Heritability for all emergence-related traits was high in both populations and environments. The cold-tolerant parent exhibited greater tolerance to the emergence in the presence of chilling stress than the cold-susceptible parent. The emergence parameters analyzed by the hill curve are presented in Tables 1, 2.

### 3.3 Soil-based indoor screening

The cold-tolerant parental lines exhibited greater tolerance, as described by the emergence parameters recorded in both cold and optimal environments, than the cold-susceptible parent. Both RIL populations showed considerable variation in observed traits. In both RIL populations, emergence, as expected, was greater (↑ Emax), faster (↓ T_50_), (↓ T_4_), and more uniform (↓ U_8416_) under greenhouse conditions compared to the cold room (Table 3). Likewise, 14-day seedlings were observed to be greener (SPAD), taller (STML), and with longer roots under ideal conditions *versus* chilling temperatures (Table 4). The narrow sense heritability was moderately high for all phenotypical traits. Under chilling stress, heritability was generally lower than under optimal conditions.

**TABLE 4:** Indoor physiological chilling response data for C1 and C2 population.

		Population C_1_					Population C_2_				
		Parents		RILs			Parents		RILs		
Traits	Location	CT19	TX430	Mean ±SD	Range	h^2^	M81E	ICSV700	Mean ±SD	Range	h^2^
SPAD	Greenhouse	25.2	20.3	21.6 ± 4.4	4.36–33.6	37	17.3	10.8	18.84 ± 3.75	0.4–12	
	Cold Chamber	8.9	0.1	5.2 ± 2.6	1.00–13.40	81.16	1.4	2.9	3.36 ± 2.45	0.4–12.0	
STML (cm)	Greenhouse	12.6	9.6	13.1 ± 3.8	3.82–27.01	79.46	15.9	11.9	18.34 ± 4.25	9.98–29.0	74
	Cold Chamber	4.9	3.2	4.0 ± 1.1	1.64–7.32	54.41	4.0	5.6	4.60 ± 1.67	0.8–10.0	
RTL (cm)	Greenhouse	10.9	8.3	11.9 ± 1.8	0.12–16.2	38	11.9	10.5	10.71 ± 1.2	7.72–13.9	50
	Cold Chamber	11.0	5.4	5.6 ± 2.0	1.25–9.64	88.03	7.1	9.6	6.42 ± 2.11	1.3–11.7	
STMWW (g)	Greenhouse	0.6	0.5	0.78 ± 0.29	0.13–1.80	57.54	0.4	0.5	0.6812 ± 0.366	0.05–1.95	50
	Cold Chamber	0.0	0.0	0.184 ± 0.76	0.01–0.39	80.02	0.3	0.2	0.1702 ± 0.11	0.01–0.55	
STMDW (g)	Greenhouse	0.1	0.0	0.137 ± 0.07	0.05–0.76	100	0.1	0.2	0.2184 ± 0.116	0.06–0.62	
	Cold Chamber	0.0	0.0	0.044 ± 0.063	0.00–0.52	17.78	0.0	0.1	0.0249 ± 0.01	0.01–0.10	
ROOTWW (g)	Greenhouse	0.5	0.6	0.748 ± 0.318	0.18–2.20	99.95	0.5	0.5	0.5686 ± 0.291	0.14–1.79	6
	Cold Chamber	0.1	0.2	0.306 ± 0.12	0.09–0.91	51.79	0.2	0.3	0.2180 ± 0.13	0.00–0.79	
ROOTDW (g)	Greenhouse	0.2	0.0	0.191 ± 0.092	0.03–0.69	58.24	0.1	0.2	0.2909 ± 0.173	0.08–1.14	
	Cold Chamber	0.1	0.1	0.0867 ± 0.039	0.03–0.19	50.26	0.1	0.1	0.094 ± 0.10	0.02–0.89	

SPAD, mean chlorophyll content (greenness) of the third leaf; STML, mean stem length in cm; RTL, mean root length in cm; STMWW, mean mass of fresh plant in grams; STMDW, mean mass of plant after 7 days of drying; ROOTWW, mean mass of fresh root; ROOTDW, mean mass of roots after 7 days of drying.

### 3.4 Correlations between controlled and field screening

**FIGURE 1:**
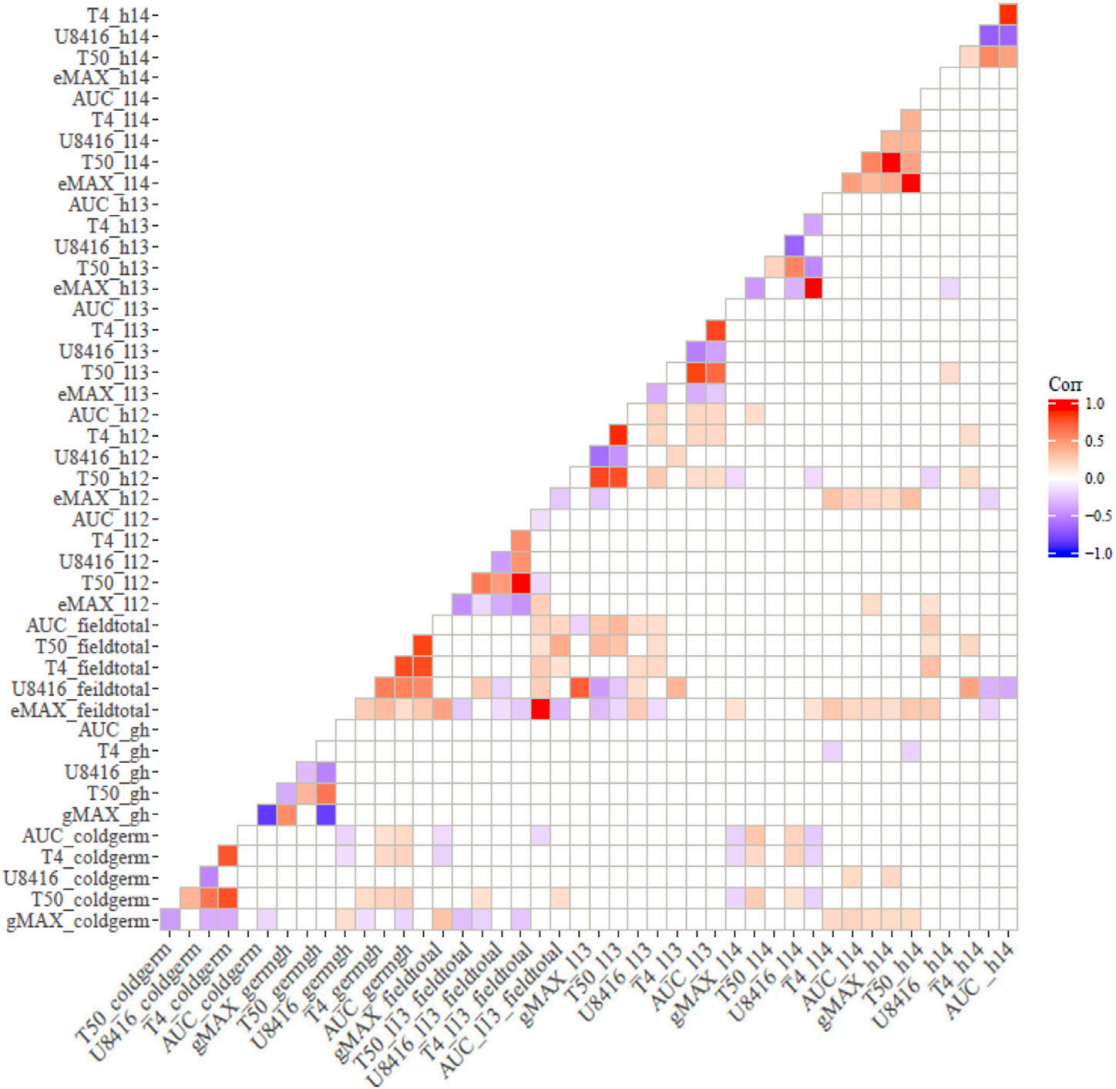
Pearson’s Correlation between indoor germination and field emergence test for CT19 x TX430 (non-significant correlations have been left blank).

**FIGURE 2:**
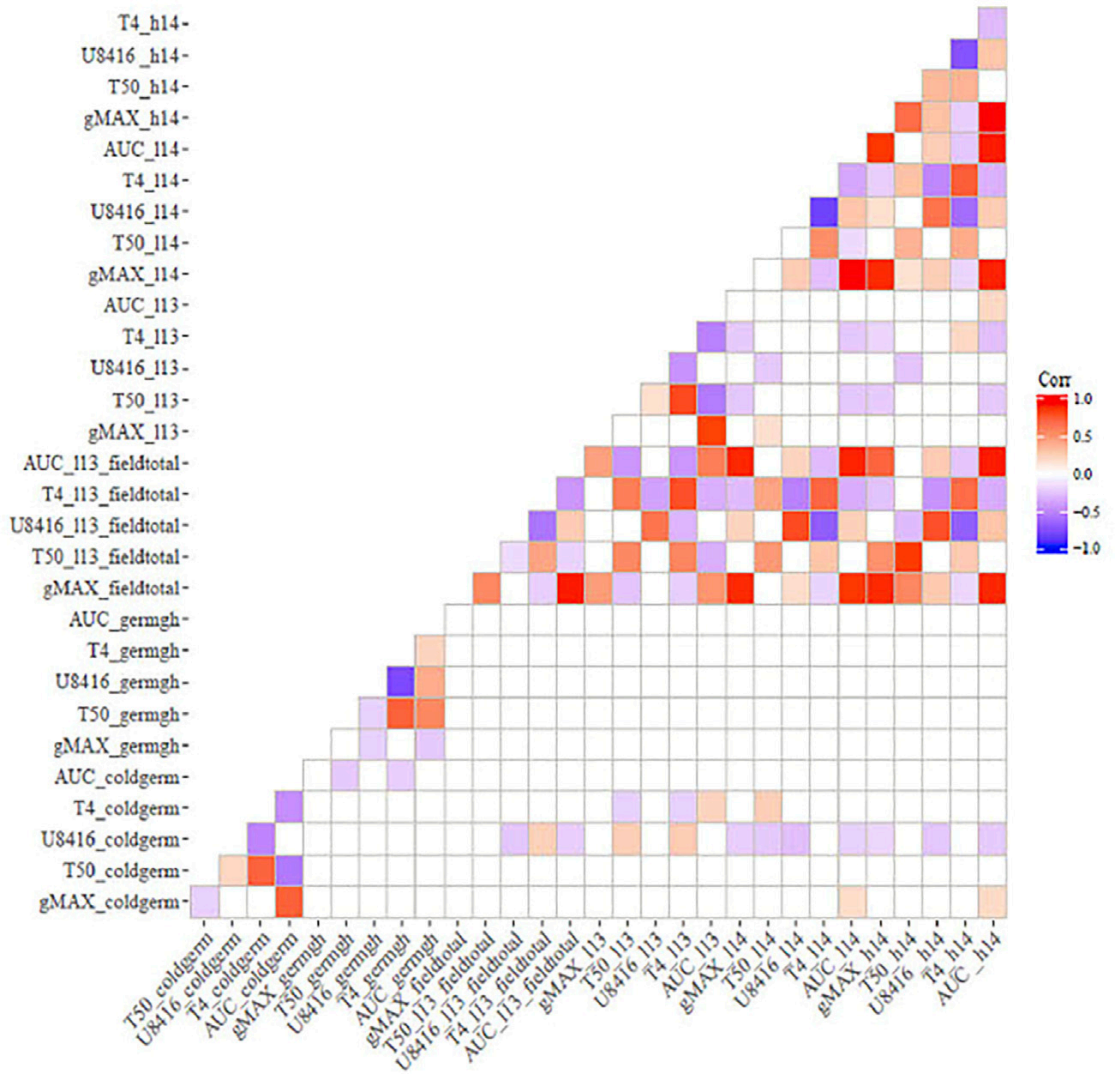
Correlation between indoor germination and field emergence test for ICSV700 x M81E (non-significant correlations have been left blank.

Correlation analysis among germination trails and field emergence screenings revealed the relationships between indoor and outdoor parameters. As anticipated, germination in optimal and chilling conditions exhibited significant correlations in both populations (Figures 1, 2). Indoor germination under chilling conditions also significantly correlated with field emergence traits—especially pooled emergence traits. While correlations were significant, the correlation coefficient was very low for both populations. Field emergence traits also correlated significantly with other field triats in both populations.

### 3.5 Linkage map

For both the C_1_ and C_2_ populations, linkage maps were constructed using GBS-SNP markers (Supplementary Figures S1, S2) with less than 30% missing data. For the C_1_ population, 464 polymorphic GBS-SNPs were mapped to 12 linkage groups. The total map length was 2080.1 cM and covered all ten chromosomes. The lengths of the individual linkage groups ranged from 57.3 cM to 295.8 cM, with an average marker density of 4.6 cM. Eight hundred seventy-five polymorphic SNP markers were mapped to 14 linkage groups (1,515.2 cM total length) in the C_2_ population. Markers covered all ten chromosomes; the linkage groups ranged from 15.03 cM to 242 cM with an average marker density of 1.8 cM per marker.

### 3.6 QTL analysis

**FIGURE 3:**
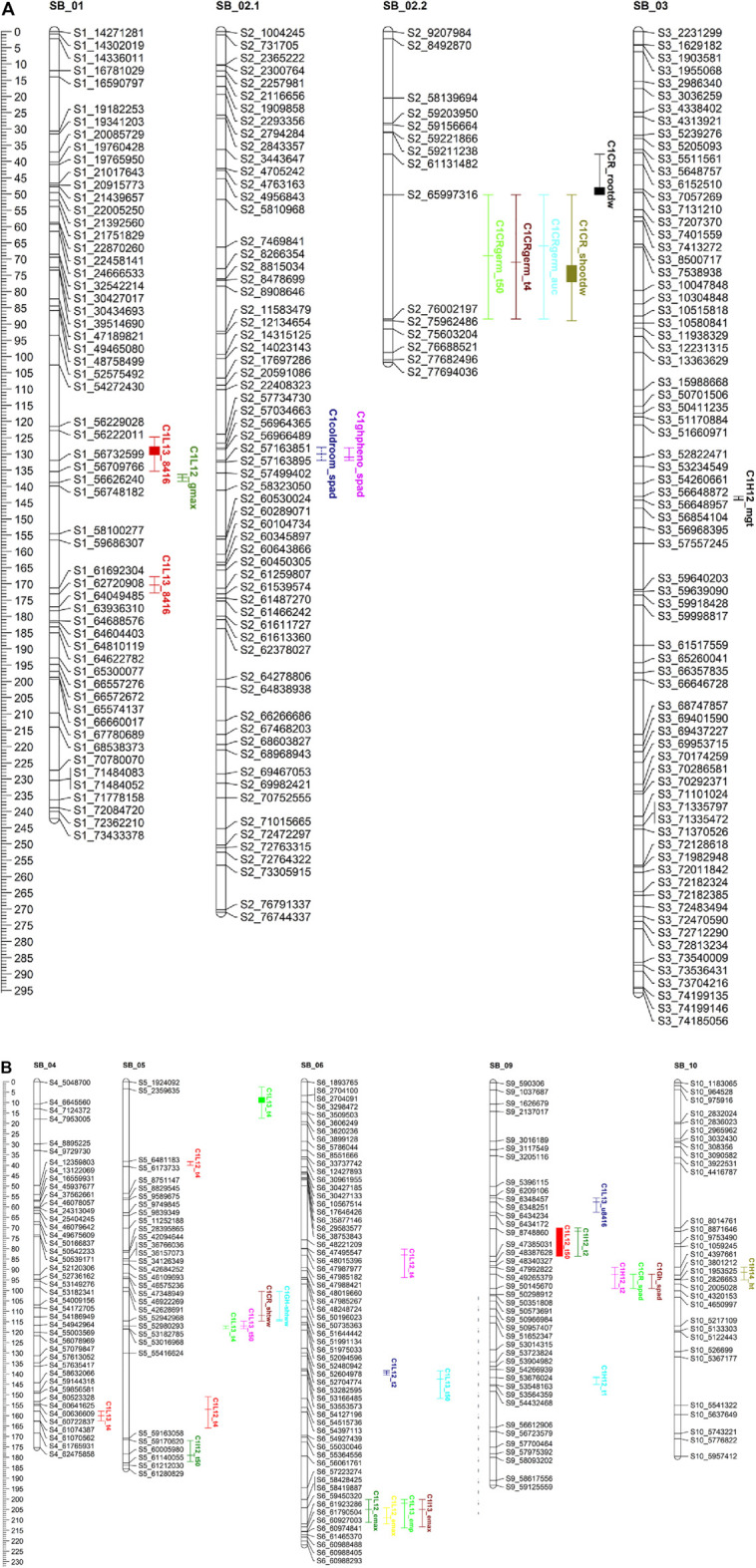
**(A, B)** QTL mapping for population C1 using 464 SNP markers with traits data obtained from the field (during 2012.2013.2014) and the combined controlkd indoor experiments. The traits considered are seedling chlorophyll content (wad). phnt height(M). Shoot wet weight (shootww). Shoot dry inter! (shootdw). and root dry weight (rootdw). Emergence percentage lemp). time to 4% emergence (T4), time to 50% emergence (T50), mean germination time (mgt), and uniformity of emergence (U8416).

**FIGURE 4:**
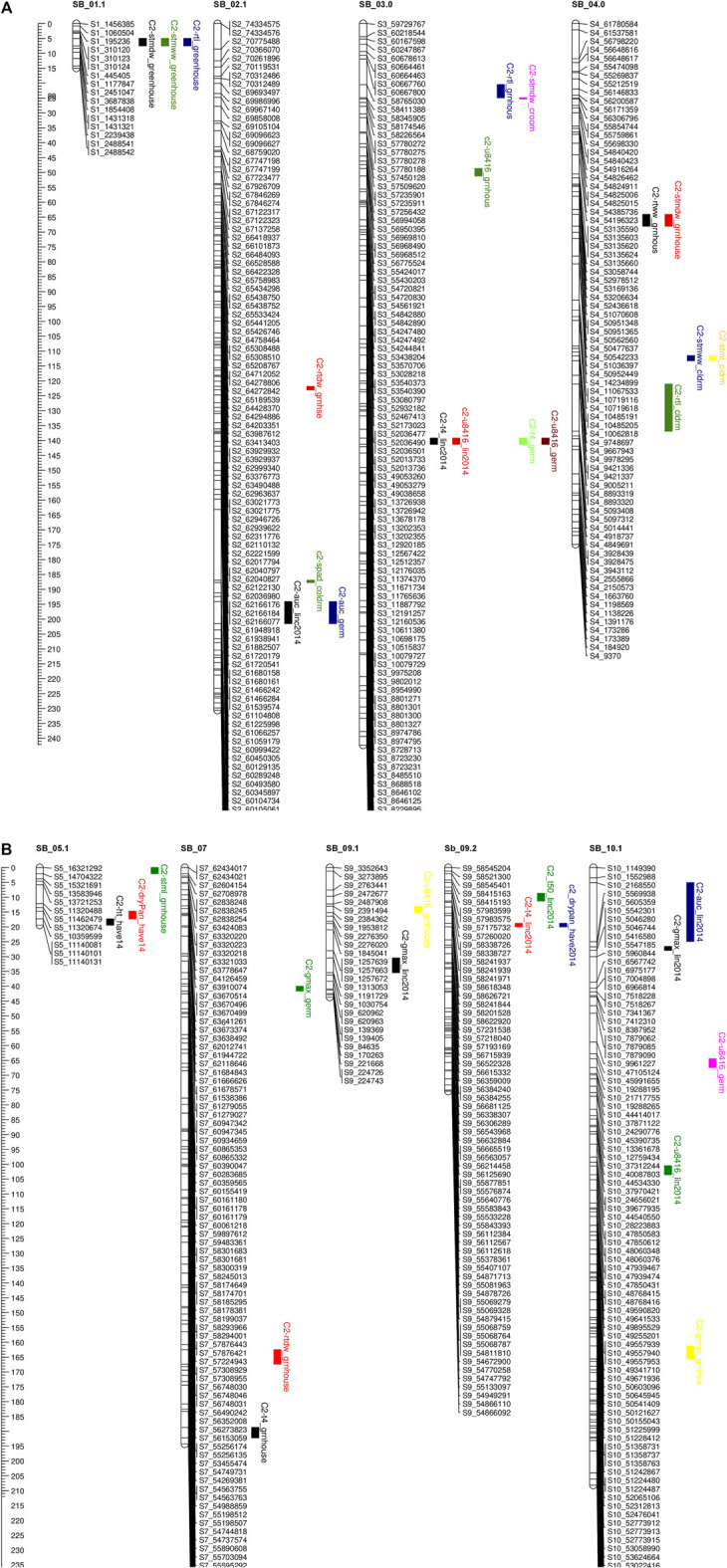
**(A, B)** QTL mapping for population C2 using 875 SNP markers with traits data obtained from the field (during 2012.2013.2014) and the combined controlled indoor experiment The traits considered and seedlling Chlorophyll content (spad). plant height (ht), shoot wet weight (shootww). Shoot dry weight (shootdw). and root dry weight (rootdw). Emergence percentage (emp). time to 4% emergence (T4), time to 50% emergence (T50), and uniformily of emerengence (U8416).

QTL mapping was carried out to elucidate genomic regions underlying seedling response to chilling. The results of the QTL analysis for all germination/emergence-related traits, along with SPAD, root and stem length, and their fresh and dry weights in both C_1_ and C_2_ populations, are highlighted in Tables 5, 6, Figures 3A,B, Figures 4A,B. QTL were considered stable if they appeared in more than one location for a specific trait and consistent if they appeared in more than 1 year/season. QTL identified are highlighted below.

**TABLE 5:** Main Effect QTL positions for C_1_ population.

								
Population	Location & year	QTL	LG	Peak	Marker interval	AE	LOD	PVE
C_1_	Lincoln 2012	C1L12_t50	SB_05	179	S5_60005980/S5_61140055	−1.0766	4.22	9.95
		C1L12_t1	SB_05	157	S5_5516624/S59163058	−2.83	3.06	22.21
C_1_	Havelock 2012	C1H12_t2	SB_09	89	S9_47992822/S9_50145670	0.9443	3.1	7.25
	Havelock 2013	C1H13_gmax	SB_09	177	S9_58093202/S9_58617556	0.026	2.46	6.43
	Lincoln 2013	C1L13_t1	SB_05	5	S5_1924092/S5_6481183	2,719	4.33	10.71
		C1 _L13_t1	SB_04	160.5	S4_60722837/S4_61070562	−0.1975	2.51	5.38
		C1L13_U8416	SB_01	132.5	S1_56222011/S1_56732599	−16.448	4.89	12.4
		C1L13_U8416	SB_01	172.5	S1_59686307/S1_561692304	12.67	2.9	7.18
		C1L13_t10	SB_05	82.5	S5_42094644/S5_36766036	−11.35	2.5	6.56
C_1_	Greenhouse	C1GH_spad	SB_2.1	132	S2_56966489/S2_57163895	−1.08	3.2	7.79
		C1GH_shtww	SB_05	114	S2_56966489/S5_52980293	−0.075	3.01	8.34
		C1_GH_spad	SB_09	89	S9_49265379/S9-50145670	0.96	2.56	6.21
		C1GH_rootl	SB_06	216	S6_60974841/S6_61465370	0.42	2.44	6.04
C_1_	Cold Chamber	C1CR_shootdw	SB_2.2	77	S2_65997316/S2_76002197	−0.11	2.76	15.45
		C1CR_rootdw	SB_2.2	50	S2_61131482/S2_65997316	0.07	2.6	5.41
C_1_	Cold Chamber	C1coldgerm_t50	SB_2.2	69	S2_65997316/S2_76002197	−21.5	2.7	19.1

Summary of main-effect QTL, positions in the CT19 x Tx430 RIL, population (n = 240) from chilling stress tolerance experiments. QTL, positions (Peak).

^a^ -LOD, flanking markers, additive effects (AE) and percent of explained phenotypic variance (PVE).

**TABLE 6:** Main effect QTL positions for C_2_ population.

								
Population	Location & year	QTL	LG	Peak	Marker interval	AE	LOD	PVE
C_2_	Lincoln 2013	C2L13_u8416	SB_03	51	S3_554424017/S3_56775524	−48.41	3.01	9.63
		C2L13_t4	SB_07	192	S7-7490519/S7_7081933	37.46	2.88	9.42
		C2L13_gmax	SB_07	39	S7_60934659/S7_6086535	−0.037	2.83	8.3
		C2L13_u8416	SB_09	15	S9_2391494/S9_2384362	−43.99	2.54	7.96
		C2L13_gmax	SB_08	0	S8_3115611/S8_306121	0.0375	2.51	8.38
	Lincoln 2014	C2C14_s	SB_06	30	S6_39193351/S6_39192899	−0.23	2.6	7.52
	Lincoln 2014	C2H14_mgt	SB_04	12	S4_567982/S4_6153781	−6.66	3.15	7.8
		C2H14_t4	SB_09	6	S9_58415193/S9_57983599	9.43	2.75	6.99
		C2H14_mgt	SB_01	8	S1_2451047/S1_3687838	6.43	2.73	7.11
		C2H14_u8416	SB_03	71	S4_53080797/S4_52932182	13.02	2.59	6.81
		C2H14_t50	SB_01	4	S1_445405/S1_1177847	6.68	2.49	6.86
C_2_	Lincoln 2013	C2L13_s	SB_09	33	S9_1313053/S9_1191729	0.053	10.25	30.34
		C2L13_emp	SB_09	36	S9_1191729/S9_1313053	0.062	5.25	12.41
		C2L13_t50	SB_09	36		2.96	4.7	11.53
		C2l13_s	SB_09	13	S9_2487908/S9_2391494	−0.3433	4.77	12.78
		C2L13_t50	SB_2.2	36	S2_69096627/S2_68759020	2.19	2.7	6.26
		C2l13_s	SB_07	60	S7-58245013/S7_58174649	−0.25	2.51	6.49
C_2_	Havelock 2014	C2H14_emp	SB_09	36	S9_56384255/S9_56681125	0.0523	2.97	6.67
		C2H14_s	SB_06	30	S6_39193351/S6_39192899	−0.023	2.5	7.7
C_2_	Greenhouse	C2GH_stmdw	SB_01	7	S1_117847/s1_2451047	0.054	4.29	26.52
		C2GH_rtdw	SB_2.2	123	S2_61680161/S2_61466242	−0.053	3.95	26.31
		C2GH_stml	SB_05	0	S5_16321292/S2_61466242	−1.77	2.85	17.33
		C2GH_rtl	SB_03	24	S3_58765030/S3_58411388	−0.5	2.8	19.1
		C2GH-rtww	SB_04	66	S4_50951365/S4_50562560	−0.095	2.68	19.27
		C2GH_stml	SB_1.2	16	S1_55581071/S1_52448063	−1.89	2.58	19.89
C_2_	Cold Room	C2CR_rtl	SB_04	131	S4_5093408/S4_5093408	−1.16	7.38	28.06
		C2CR_stmdw	SB_03	25	S3_58411388/S3_58345905	−0.004	3.49	13.52
		C2CR_rtl	SB_06	31	S6_39193351/S6_39192899	0.624	2.79	8.65
		C2CR_stmww	SB_1.2	1	S1_56849548/S1_56871988	0.033	2.51	9.07
C_2_	Germination	C2germ_t50	SB_02	69	S2_65997316/S2_76002197	−21.49	2.6	19.06
	(coldroom)	C2germ_t4	SB_02	71	S2_65997316/S2_76002197	−24.92	2.52	16.79

Summary of main-effect QTL, positions in the M81 E x ISCV700 RIL, population (n = 183) from chilling stress tolerance experiments. QTL, positions (Peak).

^a^ -LOD, flanking markers, additive effects (AE) and percent of explained phenotypic variance (PVE).

#### 3.6.1 Field experiment

In population C_1,_ ICIM detected ten additive QTL for seedling emergence and growth under chilling stress across 3 years and two environments (Figures 3A,B). In 2012, at the Lincoln site, two significant QTL were identified on SB05. One for time to 50% emergence (T_50_) and the other for time to onset of emergence (T_4_); the former explained 9.95 the latter 22.21% phenotypic variation. At the Havelock, NE site, one significant QTL for time to cessation of emergence (T_4_) was identified on SB_09, explaining 6.43 percent of the observed variation (Table 5). At the Lincoln location, in 2013, five QTL were detected. Two QTL describing 10.71% and 5.38% of the variation in onset to seedling emergence were observed on SBI-05 and SBI-04. Two QTL for uniformity of emergence (U_8416_) were identified on SB_01, one accounting for 12.4 and the other for 7.18% of the variability in uniformity. Finally, a QTL for time to 10% emergence was identified on SB_05, describing a 6.56% variation in time. At Havelock location, two QTL were observed on SB_09. One for maximum emergence percentage (Emax), describing 6.43% of the variation in emergence percentage, and the other for the area under the curve (AUC) was responsible for 6.92% variation in the area under a curve. On SBI-06, a QTL for root length was identified, describing 6.04% of the variation in length. In population C_2_, 19 additive QTL were identified for seedling emergence and growth (Table 6; Figures 4A,B). At Lincoln, NE, in 2013, two QTL for uniformity of emergence were identified. One was located on SBI-03, the other on SB_09, accounting for 9.63% and 7.96% of the variation. A QTL for time to 4% seedling emergence was observed on SB_07, which explained 9.42% of the observed differences in time to 4% germination. Two QTL describing the maximum emergence percentage was located on SB_08, accounting for 8.38% of the variation. Six additive QTL were detected for the Lincoln 2014 experiment. Three QTL for the velocity of emergence were identified, two of which resided on SB_09, accounting for 43.12% of the variability in observed velocity. The final velocity QTL was observed on SBI-07, contributing 6.46% to variability in emergence velocity. Two QTL for time to 50% germination were identified, one on SB_09 and the other on SBI-2.2, accounting for 11.53% and 6.26% variation in time, respectively. One QTL for maximum emergence percentage was identified on SB9, explaining 12.41% of the variability in emergence. At the Havelock site in 2014, two additive QTL for maximum emergence time were identified on SBI-01 and SBI-04, explaining 7.11% and 7.8% of phenotypic variation. A QTL for emergence percentage was identified on SBI-09, explaining a 6.67% variation. A velocity of emergence QTL, explaining 7.52% of the variation in the emergence rate, was identified on SBI-06. A QTL for 4% emergence was located on SBI-09, accounting for a 6.99% variation in time to 4% emergence. A QTL for uniformity was identified on SBI-03, accounting for 6.81% of the observable variation.

#### 3.6.2 Cold room experiment

In the C_1_ population, three QTL were identified in the linkage group SB_2.2. One for time to 50% germination, another for seedling shoot dry weight and seedling root dry weight. Each QTL accounted for 19.1, 15.45, and 5.45% of phenotypic variation, respectively.

Eight additive QTL were observed from the cold room experiment in the C_2_population. Two QTL for seedling root lengths were identified on SB_04 and SB_06, explaining 28.01% and 8.65% of the respective differences in root lengths. Two QTL for seedling stem dry weight were observed on SB_03, and SB_06 explained 13.52% and 9.1% of the variation in dry weight. Two QTL for seedling wet weight were detected on SB_04, and SB_1.2 accounted for 11.41% and 9.07% of the observed variation. One QTL was identified on SB_02 for SPAD (leaf greenness), accounting for 5.2% of the observed variation. Finally, a QTL on SB_04 was detected, accounting for 5.4% of stem length variability.

Five QTL were observed for germination in the cold room. One for time to 4% germination was identified on SB_03, explaining 12.2 of the variability on germination time. Two QTL for uniformity of germination (U8416) were identified on SB_10 and SB_03, accounting for 10.6% and 9.2% of the variation in uniformity observed. On SB_02, a QTL for AUC, which accounted for 8.6% of phenotypic variation, was identified. A QTL for Gmax was identified on SB_07, describing a 5.1% of the variation in germination percentage.

#### 3.6.3 Co-localization of QTL

All the recorded germination, emergence, and vigor phenotypes for both populations were identified to co-localize on unique regions across the ten linkage groups (Figures 3A,B; Figures 4A,B). These locations are similar to those identified in previous QTL mapping experiments.

## 4 Discussion

Early season chilling is a significant constraint in sorghum production in the temperate US Great Plains. To establish/expand sorghum as a viable crop, good seed germination, emergence, and high seedling vigor in the early-stage chilling condition is necessary. Cold tolerance in sorghum at the germination stage can be defined as the ability of the seeds to germinate at temperatures that are usually lower than the optimum ones, which is about 10°C. Selection of an appropriate screening temperature for cold tolerance is crucial. Too high a temperature will not allow differentiation in development among lines, and below 10°C temperature will stop all growth (Hope et al., 1992). Significant variation of cold tolerance was also reported in the literatures in commercial sorghum hybrids under controlled low temperature in the laboratory (Yu et al., 2004; Maulana et al., 2017; Marla et al., 2019, Rutayisire et al., 2021). Sorghum planted under chilling conditions typically results in reduced germination, emergence, and uneven plant stands within rows. Planting sorghum earlier in the spring will allow producers to take advantage of increased rainfall and cooler temperature, allowing for pollination and fertilization to avoid the hot and dry summer.

In this study, seed germination/emergence was evaluated in two different *S. bicolor* populations: genotypes from the respective RIL populations from a cross of tolerant and susceptible sorghum lines were used to elucidate genomic regions associated with seed germination, emergence, and seedling vigor under cold and optimum conditions. Using field and growth chambers for phenotyping allowed a greater understanding of chilling stress’s genetic complexity. QTL regions for seedling germination, emergence, and physiological traits were detected for chilling and non-chilling stress. The vast number of QTL detected further illuminates the complex and polygenic nature of seed germination, emergence, and seedling physiology under cold conditions.

### 4.1 Genetic variation in chilling tolerance and related traits

During early-season planting, sorghum seedlings may be exposed to chilling stress, which often decreases field establishment. From our study, seedling soil emergence onset, duration, and speed were hindered by chilling temperatures, indicating that environmental factors play a crucial role in seedling development. Also, the filter-based germination test proved an excellent indicator of field-based emergence under chilling temperatures. Under both chilling and optimal conditions, the cold-tolerant parents showed early onset, more uniformity, and faster germination/emergence. These parents were also observed with greater root and shoot length and biomass. These results are similar to those previously reported by Podder et al. (2020) and Salas Fernandez et al. (2014). These results suggest that germination rates may be used to screen germplasm for tolerance to early-season chilling. These chilling tolerant parents were also observed with more significant root and shoot length and biomass than non-tolerant parents. This was similar to what was observed by Moghimi et al. (2019), suggesting that seedling root and shoot vigor are indicators of chilling tolerance. The high variability in traits between the genotypes in both populations demonstrates the possibility of breeding to improve cold tolerance. The high heritability estimates allow for the possible selection of high cold tolerance. The C_1_ and C_2_ populations have varying degrees of cold tolerance, which can be exploited to improve sorghum to early season chilling temperatures.

### 4.2 SNP markers and linkage mapping

Most previously constructed linkage maps for intra-specific sorghum have utilized SSR markers. The identification of polymorphic SSR markers is both time and labor-intensive. As a result, previous studies (Bekele et al., 2014; Burow et al., 2010; Fiedler et al., 2016, 2014; Knoll et al., 2008). have been limited to a few hundred markers at best. In recent years, next-generation sequencing (NGS) has effectively constructed high marker-density genetic maps with respect to time, labor, and costs. A GBS approach was utilized to produce a high-density, low-cost genetic map (Gore et al., 2014), allowing the identification of SNPs and genotyping at the same time. We identified a total of 1,339 novel SNPs between the two populations. In comparison to previous GBS studies (Gore et al., 2014; Zheng et al., 2011), in other plant species, we identified fewer SNPs in the present experiment. This may be attributed to the intrinsic disadvantage of GBS-SNP: a large amount of missing data due to narrow sequencing depth.

Additionally, other SNPs may have needed to be recovered in attempts to capture high-quality data by removing SNPs with >30% missing data. Sequencing errors may have also expanded the genetic distance between markers in the two linkage maps. These extended regions may hinder the discovery of QTL in such regions (Kumawat et al., 2012). Compared to previous studies, our linkage maps fell into the ranges of those previously published (Ejeta and Knoll, 2007; Burow et al., 2010). In the C1 population, chromosomes Sb02 and Sb08 and Sb01, Sb05, Sb09 (Figures 3A,B), and Sb10 in the C2 (Figures 4A,B) populations were fragmented into two linkage groups. Hiremath et al. (2012) reported linkage group fragmentation resulting from high-density genetic mapping and its lack of impact on the fidelity of QTL mapping. From allele analysis, the need for splitting linkage groups was attributed to distorted allele frequencies in the Sb02 (C_1_), Sb01 (C_2_), and Sb10 (C_2_). Large blocks of homogeneity between parental alleles caused other fragments.

### 4.3 QTL associated with cold tolerance

The four parental lines’ early season germination and seedling growth vigor varied considerably when exposed to chilling temperatures. Unlike other DNA markers, SNPs allow for the development of gene haplotyping systems (Rafalski, 2002), mainly if several defined haplotypes exist in the region of interest. Due to the absence of allelic ambiguity, co-dominant SNPs are more informative than dominant markers (Kumar et al., 2012). Unfortunately, to our knowledge, no QTL studies utilizing SNPs for early-season chilling tolerance in sorghum exist. For QTL mapping studies, populations of 100–500 individuals are suggested. Thus, the 189 genotypes for C_1_ and C_2_ were acceptable for distinguishing QTL, conferring chilling tolerance and vigor traits.

Previous mapping experiments have identified several loci contributing to early-season chilling tolerance in sorghum. Knoll et al. (2008) identified one QTL for low-temperature germination on SB_03 and two for early/late season emergence, one on SB_01 and the other on SB_02. Burrow et al. (2010) uncovered four QTL: one on SB_02 for germination in both cold and optimal conditions, one for field emergence on SB_09, and two on SB_01 for late field emergence. Bekele et al. (2014) identified three QTL for cold germination: one on SB_01, one on SB_03, and one on SB_06 (Fiedler et al. (2012) reported three QTLs for emergence percentage on SB_01, two on SB_03, and one on both SB_06 and SB_07. Finally, in an association experiment, Upadhyaya et al. (2015) detected a single QTL for cold germination on SB_07. Moghimi et al. (2019) reported markers for germination on SB_02, SB_03, SB_08, and SB_09. Emergence related markers were also detected on SB_04 and SB_07. Upadhyaya et al. (2015) detected a single QTL for cold germination on SB_07. Moghimi et al. (2019) reported markers for germination on SB_02, SB_03, SB_08, and SB_09. Emergence related markers were also detected on SB_04 and SB_07.

The complex and polygenic nature of early-season chilling tolerance was confirmed by the number of QTL detected. Sixteen and 39 QTL were identified in the C_1_ and C_2_ populations (Figures 3A,B; Figures 4A,B). Of these, four QTL in the C_1_ and three QTL in the C_2_ population have major effects (PVE 14%). Despite the different environmental settings and many genotypes, several QTL were detected across multiple years and environments. QTL detected across multiple environments are considered stable and may prove beneficial for marker-assisted plant breeding. Regions with co-locating QTL possibly housed many tightly linked genes conferring chilling tolerance and seedling vigor-related traits with pleiotropic effects. SNP’s linked to the QTL identified in this study will be used as selection tools in breeding early season vigor and cold tolerance from the cold-tolerant parents into elite sorghum breeding lines. Validation of the effects of these molecular markers across other populations and environments will be an additional, but very important, step in developing a marker-assisted breeding effort. SNP’s which can be further validated will be introgressed into elite breeding lines, which can then be evaluated in hybrid combinations.

### 4.4 QTL for physiological traits related to cold tolerance

Several seedling vigor traits were evaluated under chilling and optimal conditions to identify genomic regions associated with seedling vigor. Six QTL regions in the C_1_ population and 18 QTL for the C_2_ population were identified, confirming the polygenic nature of seedling vigor traits. Of the QTL identified, one major QTL was identified in the C_1_ population, and three major QTL for the C_2_ population. In both populations, the majority of the seedling vigor QTL were centered on SB_01, SB_02, SB_03, SB_04, and SB_05, which coincided with previously mentioned studies.

On chromosome on Sb-01, two major stem-related QTL were identified in the C_2_ population, one for dry stem weight under optimal conditions where the M81e alleles conferred extracellular mass to seedlings’ stem. The other QTL identified stem length under optimal conditions. Investigating the intervals spanning these QTL, numerous putative genes were identified (Supplementary Table S2). Genes in the underlying stem dry weight QTL interval included a response to cytokinin and desiccation (Sb01g001660). Genes identified in the underlying stem length QTL interval included a response to growth hormones (Sb01g031060; Sb01g030930; Sb01g031050), a response to cold (Sb01g031870), *etc.*

SB_02 contained one major QTL for shoot dry weight, in optimal conditions, in the C_1_ population (Figures 3A,B) and one major QTL for root dry weight in the C_2_ population (Figures 4A,B). The QTL for shoot dry weight conferred a reduction in stem mass. This QTL co-localized with QTL for germination and emergence in chilling temperatures, as described earlier. The underlying intervals contained the same putative genes. Under optimal conditions, the QTL for root dry weight led to a reduction in root mass in seedlings possessing alleles. The interval spanned by the QTL contained putative genes for response to abiotic stress (Sb02g026490; Sb02g026500) and membrane functions (Sb02g026380; Sb02g026460).

Under optimal conditions, a major QTL for root length was discovered on SB_03 in the C_2_ population (Figures 4A,B). The allele led to a reduction in the length of seedling roots. Root length QTL was previously mapped to SB_03 (Bekele et al., 2014). However, these were different intervals we reported earlier. In our QTL interval, putative genes are highlighted in the Supplementary Table S2. Two major QTL for dry root weight and root length were detected on SB_04. The alleles at both QTL reduced the seedlings’ stem weight and root length. Moghimi et al. (2019) also reported a two QTLs for root biomass in the same region. One QTL for stem length under optimal conditions was identified on SB_05. The QTL for root dry weight has not previously been detected; upon an investigation of the underlying interval, some putative genes are highlighted in Supplementary Table S2. The QTL for root length co-localized with a QTL previously identified by Bekele et al. (2014).

## 5 Conclusion

The study was conducted based on the hypothesis that sorghum plants can tolerate low temperatures during germination and must contain genes that can be mapped that contribute to this tolerance compared to plants that will not germinate under those conditions. Early season chilling stress significantly limits sorghum productivity in the Great Plains. Chilling stress is a complex trait to study. While a great deal of the physiological and biological responses to chilling is well characterized in sorghum, the genetics underlying chilling tolerance are still being gleaned. The present study has added to understanding the genetics and physiology governing early season chilling tolerance through elucidation of QTL for germination, emergence, and seedling vigor and assigning functions to said QTL. The identified areas may serve as starting points for the enhancement of molecular breeding programs by allowing for an improvement in selection efficiency through a selection of QTL that allows for sorghum to improvegermination, emergence percentage, and respective rates while decreasing the time needed for both in the presence of chilling temperatures. As more NGS-based mapping and association studies are conducted, we anticipate the fine mapping of major QTL hubs and the discovery of new areas conferring chilling tolerance to sorghum. The results provide important new insights for adaptive crop breeding in the face of climate change and the expansion of sorghum production to different regions. This will facilitate sorghum from being a “crop of the future” to transforming into a real-life important agricultural alternative. Further studies are needed to confirm these QTLs by using near isogenic lines (NILs) and backcrossing methods. With the identification of increasing numbers of favorable alleles at QTLs for cold tolerance by QTL analysis, the pyramiding approach and MAS strategy could become a promising approach for improving cold tolerance.

## Data Availability

The authors acknowledge that the data presented in this study must be deposited and made publicly available in an acceptable repository, prior to publication. Frontiers cannot accept a manuscript that does not adhere to our open data policies.
