# Supplementary material for: Novel QTL for chilling tolerance at germination and early seedling stages in sorghum
Source: Front Genet. 2023 Mar 15;14:1129460. doi: 10.3389/fgene.2023.1129460 (PMC10052408; doi:10.3389/fgene.2023.1129460)
Supplement: Supplementary file 1 [file DataSheet1.pdf]

Supplementary Figure 1: Genetic linkage map generated using single nucleotide polymorphism markers in 240 sorghum lines from the recombinant inbred line population CT19 x TX430. A total of 464 markers were mapped into 12 linkage groups, with a total linkage distance of 2080.1 cM

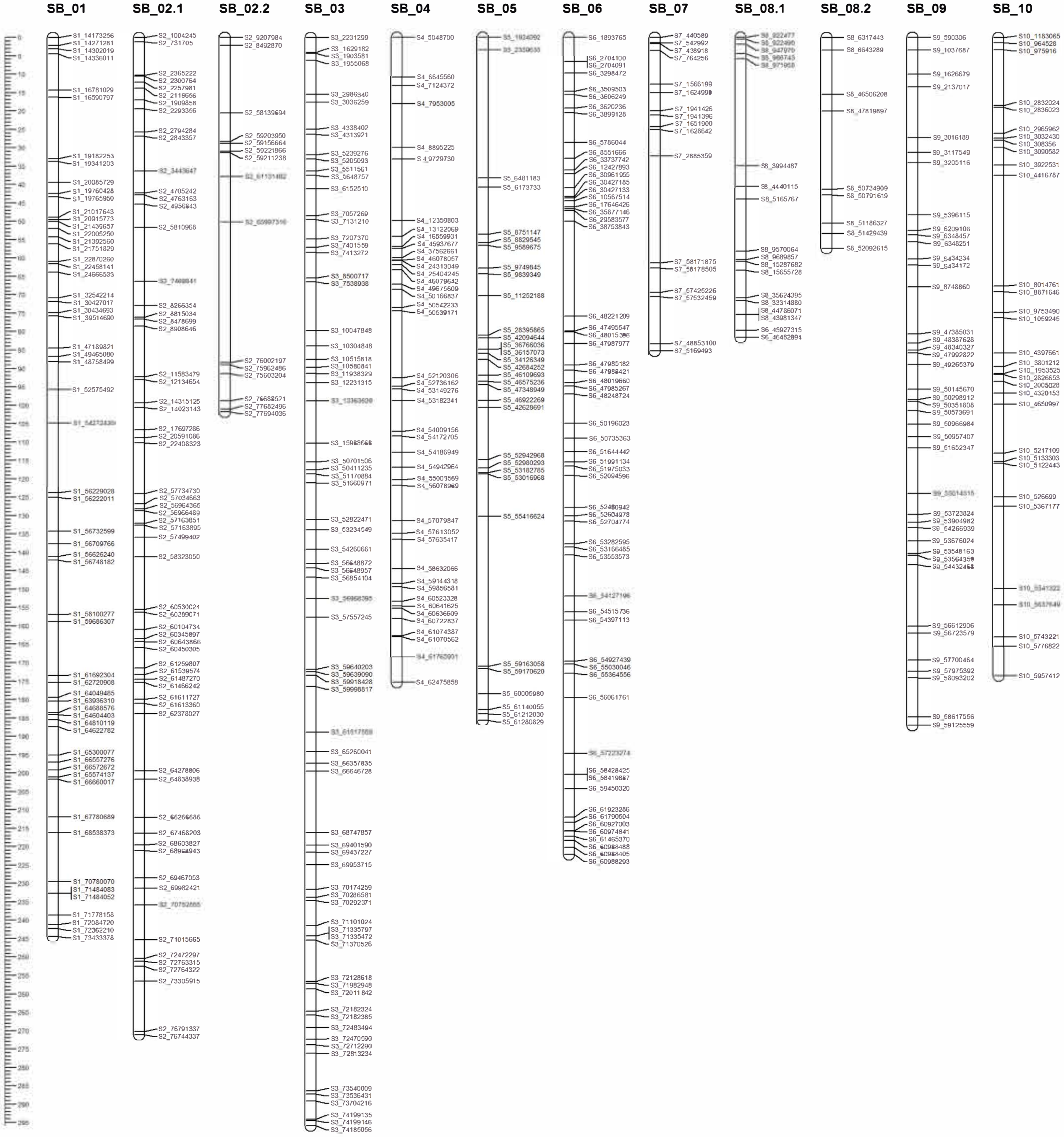

Supplementary Figure 2: Genetic linkage map generated using single nucleotide polymorphism markers in 183 sorghum lines from the recombinant inbred line population M81E x ISCV700. A total of 875 markers were mapped into 14 linkage groups, with a total linkage distance of
1515.2 cM

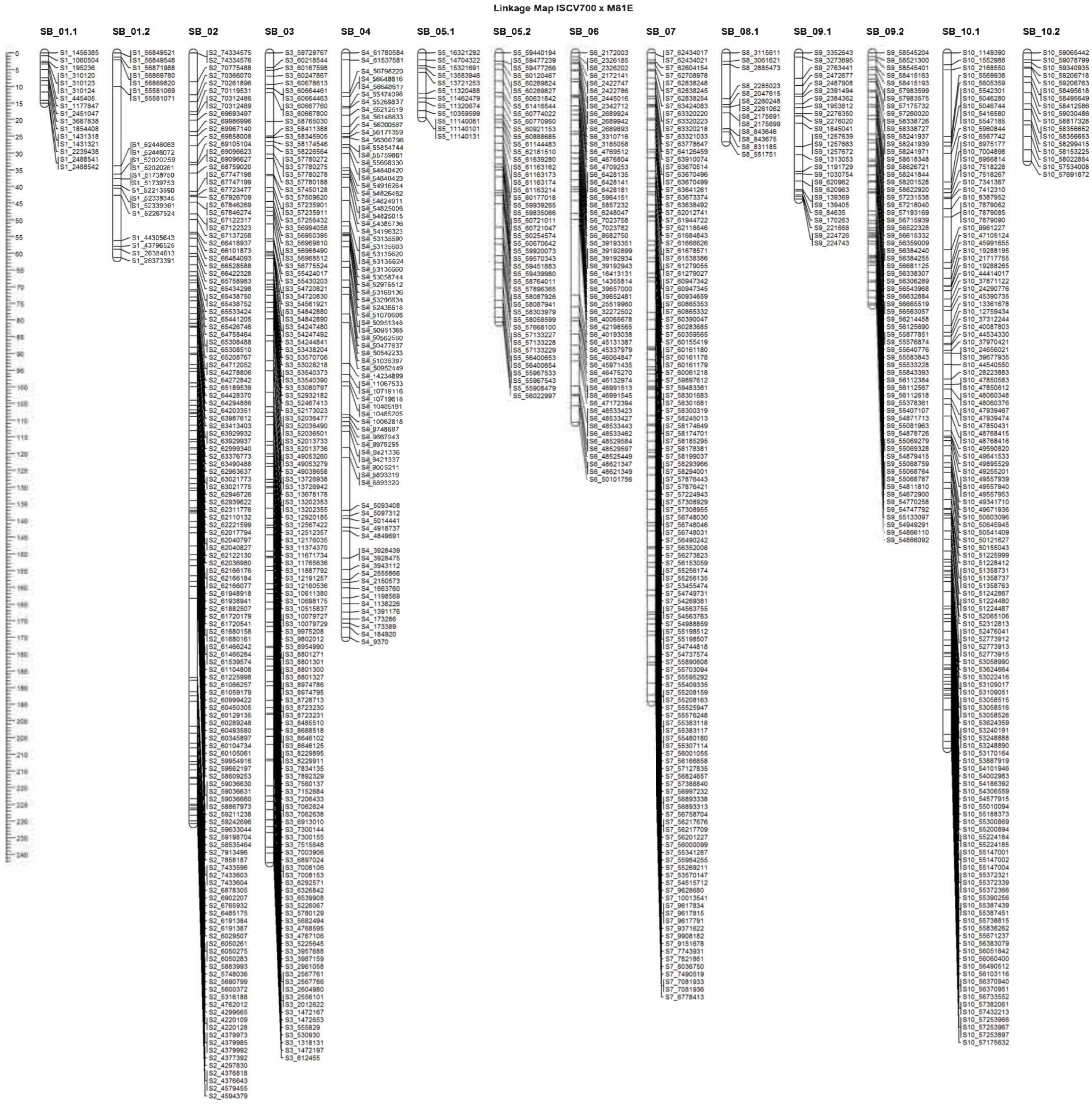

Supplementary Table 1: Temperature parameters during field trials from 2012 to 2014.

Year	Average High (°C)	Average Low (°C)	Total Precipitation (cm)	Snowfall (cm	Snow Depth (cm)
**2012**	24.4	8.9	13.23	-	-
**2013**	18.8	5.3	12.26	5.94	4.52
**2014**	15.7	7.23	13.58	-	-

Supplementary Table 2: Genes in support intervals with gene ontology terms

Chromosomes	Total # of Genes	Locus	Physical Position	Annotation
SB_01	183	Sb01g030400	52848006-52852790	response to biotic stimulus
		Sb01g030460	52919687-52920255	vacuolar membrane
				cytosol
		Sb01g030580	53148491-53150526	plasma membrane
		Sb01g030650	53243409-53246781	response to abscisic acid
		Sb01g030670	53251985-53254003	biological_process
				plasma membrane protein phosphorylation
				response to abscisic acid
				abscisic acid-activated signaling pathway
		Sb01g030810	53440691-53441773	seed germination
		Sb01g030930	53534470-53535622	lateral root development
		Sb01g031220	53797928-53802472	response to growth hormone
				lateral root development
		Sb01g031280	53848293-53852473	response to growth hormone
				cytosol
		Sb01g031660	54235597-54241230	protein phosphorylation
				response to brassinosteroid
				trehalose metabolic process
		Sb01g031870	54605448-54606683	trehalose catabolic process
				trehalase activity
				lipid biosynthetic process
		Sb01g032440	53327870-55329946	response to light stimulus
				chloroplast
SB_02	733	Sb02g031440	66379717-66382726	chloroplast stroma
		Sb02g031700	66605924-66609511	plasma membrane
		Sb02g031710	66610668-66611128	response to desiccation
		Sb02g031720	66615420-66615833	response to cold
		Sb02g031730	66616928-66617649	chloroplast
		Sb02g031740	66618719-66619443	regulation of meristem growth leaf formation
		Sb02g031760	66625890-66626288	oxidoreductase activity
		Sb02g031770	66628365-66629115	response to stress
		Sb02g031780	66631001-66631441	response to auxin
		Sb02g031790	66632690-66633130	response to auxin
		Sb02g031800	66633786-66634884	response to auxin
		Sb02g031810	66638301-66638735	response to auxin
		Sb02g031820	66639849-66640280	response to auxin
		Sb02g031830	66641296-66641655	response to auxin
		Sb02g031840	66642927-66643358	response to auxin
		Sb02g032262	67036016-67036222	response to auxin
		Sb02g036250	70669176-70671026	response to auxin
		Sb02g040260	74254800-74256992	response to auxin
		Sb02g040650	74565147-74566293	response to auxin
		Sb02g041470	75277628-75283120	response to auxin
SB_04	10	Sb04g021660	50788863-50794942	response to auxin
		Sb04g021700	5082916-50832506	response to auxin
		Sb04g021730	50853425-50855679	response to oxidative stress
		Sb04g021740	50857761-50860834	response to water deprivation
		Sb04g021790	50903442-50907733	response to cold
				response to desiccation
				response to osmotic stress
				endoplasmic reticulum
				catalytic activity
				biological_process
				carbonate dehydratase activity
				polyamine biosynthetic process
